# The Effect of Electron Spin-Dependent Polarizability on Protein Activity

**DOI:** 10.3390/biom15060830

**Published:** 2025-06-06

**Authors:** Gilad Haran, Ron Naaman

**Affiliations:** Department of Chemical and Biological Physics, Weizmann Institute of Science, Rehovot 7610001, Israel; gilad.haran@weizmann.ac.il

**Keywords:** polarizability, spin, chirality

## Abstract

In recent years, it has been established that electron transport through a chiral system depends on spin. In several studies, it has further been established that charge polarization in proteins may affect their activity and, specifically, that this polarization is electron spin-dependent. Here, we review experimental methods that enable the spin dependence of protein polarizability to be recorded and describe results from several studies that indicate the importance of spin in controlling the reactivity of proteins. We conclude by suggesting why this spin dependence may be of importance and discussing how future studies might explore pathways within proteins by which polarizability affects protein reactive sites.

## 1. Introduction

Polarizability reflects the response of a system to an electric field applied to it. Since the polarizability of a system depends on the direction of the electric field relative to its frame of reference, it is described by a tensor. Further, it is frequency-dependent. Namely, if the electric field applied to the system is of low frequency, there is time for slow organization to take place, and the reorganization includes the motion of nuclei. However, for frequencies above that of vibrations, only electrons in the system can respond.

Protein molecules are subject to effective electric fields whenever they interact with any other molecules, be it proteins, solvents or ligands, as well as when they change their conformation [1,2]. One usually considers the fixed-charge groups of proteins when calculating their polarizability. Such a calculation is likely to be accurate in cases where the electric field incurred by a protein is either permanent or modulated slowly, giving charged groups ample time to respond. It is important to appreciate that in the low-frequency electric field modulation regime, the electrons in the proteins also contribute to their polarization. However, proteins may also be affected by quickly modulated electric fields, in which case **only** electrons can respond. For example, the process of ligand docking to a protein involves a sequence of adsorption–desorption events, including initial diffusion, the approach of the ligand toward the protein, the formation and breaking of individual hydrogen bonds and van der Waals interactions, the local conformational fluctuations of amino acid side chains and the displacement of water molecules from binding sites [3,4,5]. Some of these phases can be very fast, even on the picosecond timescale. While various groups in the protein may be able to respond to slower processes, essentially only electrons can respond to the fastest ones.

The case of electron/proton transfer through proteins is also relevant to the distinction between electronic and nuclear polarizability. These charged particles, upon moving through a protein molecule, generate a polarization that depends on their location during their path. Because their motion is relatively fast, it is mainly the electrons of the protein that can respond and contribute to its (high-frequency) polarization. Later, following the charge transfer process itself, the nuclei may also react to the change in the charge distribution of the system.

What is the role of polarizability in protein function? Polarizability can make two types of contributions. The first is promoting changes in the conformation of a protein. Conformational transitions can result from the motion of charged groups but also from the motion of electrons, which may increase or decrease internal interactions within the protein by affecting bond strengths and electrostatic interactions. The second contribution of polarizability is its effect on the interactions between proteins and their ligands. The motion of charges induced by polarizability effects may modulate electrostatic interactions, changing them in a different manner than they would be by fixed electric charge distributions. Changes in the electronic distribution of a protein may occur due to, e.g., the internal motion of the protein, its interaction with solvent, and its interaction with ligands. Hence, polarizability effects are not the exception but rather a common mechanism.

The importance of polarizability for protein function and dynamics has been understood for a long time, particularly in the context of molecular dynamics (MD) simulations of proteins. Indeed, early on, Warshel and Levitt used a polarizable force field in their simulation of the role of electrostatics in lysozyme [6,7]. More recently, polarizable force fields that can be used within popular MD packages have been developed. Several approaches have been used, including, e.g., AMOEBA, based on induced multipoles [8], or the Drude oscillator model, which involves attaching a charged auxiliary particle to an atom via a harmonic spring [9].

The application of these force fields in simulations remains a challenge. Standard MD simulations can tackle the question of configurational polarizability, namely that part of the polarizability that is only due to the motion of protein groups in response to charges while not involving electronic motion. Takano and colleagues used such an approach to probe the role of configurational polarizability in inducing a conformational change in myosin in response to the binding of the nucleotide adenosine triphosphate (ATP) [10]. Impressively, the dielectric response to ATP binding leads to signal transmission through the protein that the authors called ‘dielectric allostery’. The reader is reminded that allosteric effects involve the modulation of the function of a protein through the binding of a small molecule or another protein at a location far away from the active site [11,12,13]. Classical allosteric mechanisms involve protein conformational changes [14,15], and more recently, it has been shown that changes in conformational dynamics may also lead to allosteric effects [16]. The proposed introduction of allostery due to electrostatic interactions paves the way for a general role for such interactions at various frequencies in protein function, as will be further discussed below.

Polarizability effects are important when assessing the role of electrostatic interactions at the active sites of enzymes. In fact, it is well established that electrostatic effects play an important role in enzyme catalysis. Warshel and coworkers championed the idea that charge preorganization at the active sites of enzymes is important for the reduction in transition-state free energy during an enzymatic reaction [17]. Recently, it has been shown experimentally that charges within the active site of an enzyme, ketosteroid isomerase in this case, are indeed preorganized so as to contribute a significant portion of the catalytic effect of the enzyme [18]. While only static charges have been invoked in these works, it has been suggested that polarizability is also essential for the activity of proteins; in particular, it was proposed, based on simulations, that polarizability significantly affects electron transfer in the protein cytochrome C [19].

Recently, it has been established that electron transport through chiral systems, including proteins, is spin-dependent. Namely, for a chiral system with a given handedness, electrons with one spin state move more efficiently through it than those with the other spin state. For one handedness, the electron spin is aligned parallel to its velocity, while for the other handedness, the spin is aligned anti-parallel to the velocity [20]. This effect is called chiral-induced spin selectivity (CISS) and results from the spin degree of freedom of the electron being coupled to its linear momentum when moving within a chiral system [21,22,23]. As a result of the CISS effect, the transport of electrons through proteins is efficient, since backscattering requires changing spin direction, which is a forbidden, or partially forbidden, process. 

Numerous studies have been performed on spin-dependent electron transfer through various proteins such as photosystem I [24] and D-glucose oxidase [25], bacterial cell surface multiheme electron conduits [26], and additional systems. Electron motion through a protein includes paths that are not linear and therefore require exchanging momentum with the molecular frame (nuclei), so that the electron can follow the chirality-induced curvature of the electrostatic potential [27,28]. Indeed, it has been shown that the motion of the electrons is coupled with low-frequency chiral vibrations that carry angular momentum and therefore enable transport, which implies that electron transfer through proteins is likely to be efficient at room temperature [29].

## 2. How to Directly Measure Spin-Dependent Polarization

Several methods have been developed for probing the role of spin-dependent polarization effects in proteins. We focus first on how the effect of CISS on proteins can be probed directly by observing charge transport using the Hall effect or a conductive atomic-force microscope (AFM).

**Figure 1:**
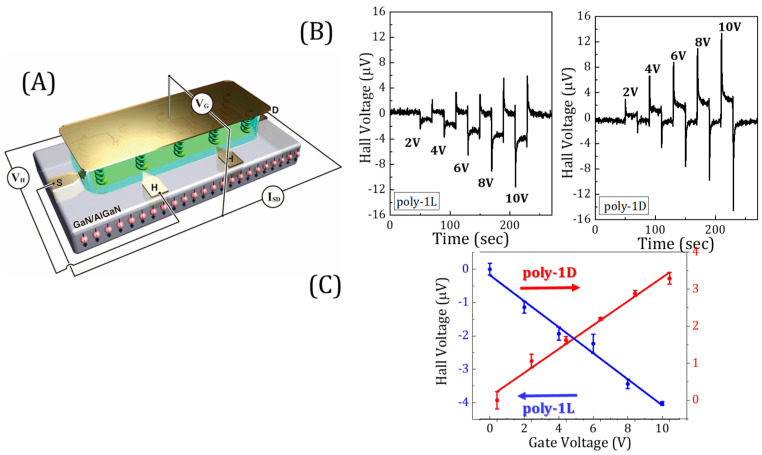
(**A**) A Hall device through which current is flowing between the source (S) and drain (D) electrodes. The Hall voltage is measured by electrodes located perpendicular to the current. The polymers poly(4-ethynylbenzoyl-l-alanine decyl ester) (poly-1L) [14] and poly(4-ethynylbenzoyl-d-alanine decyl ester) (poly-1D) are adsorbed on the device, and a gate is placed at some distance from the surface of the device. The device is immersed in solution, and the gate is insulated from the solution. When an electric potential is applied between the gate and the device, a Hall signal is measured due to voltage-induced spin polarization in the protein molecules. (**B**) The Hall signal as measured when the gate is biased at various voltages. (**C**) The Hall voltage as a function of the gate voltage for monolayers of poly-1L (blue) or poly-1D (red). The figure was copied with permission from reference [31].

In a common Hall device, a magnetic field is applied perpendicular to the current flow direction on a surface, leading to an electric potential in a direction that is perpendicular to both. We developed a different application of the Hall device that does not require applying an external magnetic field. In this application, chiral molecules, including proteins, are adsorbed on a conducting substrate. When spin-polarized electrons are injected into the substrate from these chiral molecules, the current flowing through the device is deflected so that an electric potential can be measured perpendicular to the flowing current in the absence of a magnetic field (see Figure 1A) [30]. The Hall device can be embedded into an electrolyte solution, and an electric field can be applied between a gate electrode and the device. As a result of this electric field, the protein is polarized, and if the polarization is spin-dependent, a Hall signal is measured since spin-polarized charge is exchanged between the Hall device and the protein (Figure 1B). In Figure 1C, the Hall potential is presented as a function of the gate potential when the polymers arepoly(4-ethynylbenzoyl-l-alanine decyl ester) (poly-1L) [14] and poly(4-ethynylbenzoyl-d-alanine decyl ester) (poly-1D) are adsorbed on the surface [31].

In an additional method, proteins are attached to a magnetic substrate and the current through the proteins is measured, using a conducting AFM tip, as a function of the direction of the magnetization of the substrate. An electric field is applied between the substrate and the AFM tip, and the current is measured when the magnetization of the substrate is aligned so that either its North or South pole is pointing towards the protein. The difference in current measured for the two orientations of the substrate’s magnet is an indication of the preferred spin polarization for electrons conducted through the protein [32].

## 3. Studying the Effect of CISS on Protein Function

In recent studies, it has been demonstrated that charge reorganization within a protein can serve as a novel allosteric signal. Further, it has been shown that the CISS effect can have a significant impact on the functionality of proteins, with a particular emphasis on protein–protein association reactions but also on enzymatic activity. In general, in such experiments, molecules are attached to a substrate to ensure alignment between spin injection and the protein frame of reference. However, recent studies in which spin-dependent transport was measured by EPR demonstrated the ability to follow the process even when molecules are dissolved in solution [23], a setting that is likely to be used for proteins in the near future.

### 3.1. Protein–Protein Association Reactions

**Figure 2:**
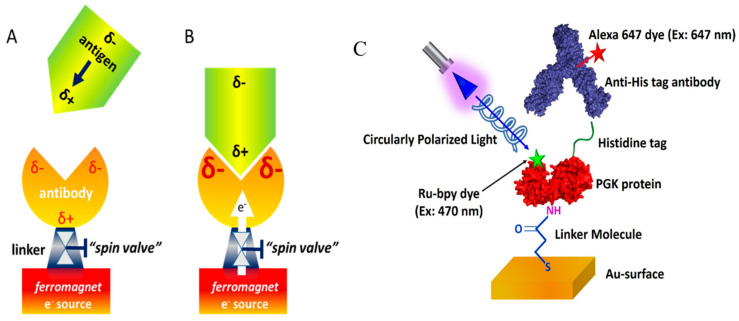
(**A**) The interaction of an antigen protein with an antibody is probed here, with a particular emphasis on the role of electrostatic interactions, both static and induced. When an antigen with a dipole moment approaches the antibody, it induces charge reorganization in the antibody. δ- and δ+ denote negative and positive charge accumulation, respectively. (**B**) The rate at which the antigen binds to the antibody depends on the amount of charge that can be accumulated at the binding site. This amount depends in this experiment on the ability of charge to move from the ferromagnet into the antibody. The rate of this flow is determined by a “spin valve”, which is based on the CISS effect (see text) and is dependent on the direction of the spin and the handedness of linker molecules attached to the ferromagnet. (**C**) A protein to which a dye is attached at a well-defined position is adsorbed on a substrate and is exposed to another protein or a ligand in the solution. The attachment of the species from the solution is monitored by their fluorescence while the protein is excited by a laser whose light is circularly polarized. The figure was copied with permission from reference [33].

The interaction between two proteins, or a ligand and a protein, typically involves an electrostatic component. Among the electrostatic terms, there is also the induced dipole, which is proportional to the polarizability of the systems. Using the CISS effect, we probed the role of induced dipoles in the association reaction of two proteins. In this experimental configuration, protein molecules were attached to a ferromagnetic substrate in a well-defined orientation, and the kinetics of their association with soluble protein molecules were monitored as a function of the direction of a magnetic field, which pointed either towards or away from the surface. If the molecular polarizability was spin-dependent, for one direction of magnetization, charge could be exchanged between the surface and the protein so that the effective polarizability would be made larger, while for the other magnetization direction, charge exchange would be impossible and therefore the effective polarizability would be smaller (Figure 2A,B). This modulation could affect the kinetics of protein–protein association, making it slower or faster, which we examined. In Figure 2C, a schematic diagram of the adsorption configuration of the protein and the experimental system is shown.

To observe this phenomenology, we performed the following experiment [33]: A histidine tag (His-tag) was inserted into the protein ClpB either on its N terminus or C terminus. An anti-His tag antibody was tethered to a gold surface deposited on a ferromagnetic layer. By changing the magnetization of the ferromagnet, we effectively created a ‘valve’ that controlled the ability of charge to flow from the surface to the antibody via the CISS effect, as discussed above. When charge flow was facilitated, the binding of ClpB through its His-tag to the antibody was modulated, which was attributed to a polarizability effect leading to the accumulation of charge at the binding site of the antibody.

Importantly, this experiment established that charge reorganization following the injection of charges at a particular site of a protein can modulate its activity remotely from the injection site. This effect was therefore called charge-reorganization allostery.

### 3.2. Protein–DNA Association

**Figure 3:**
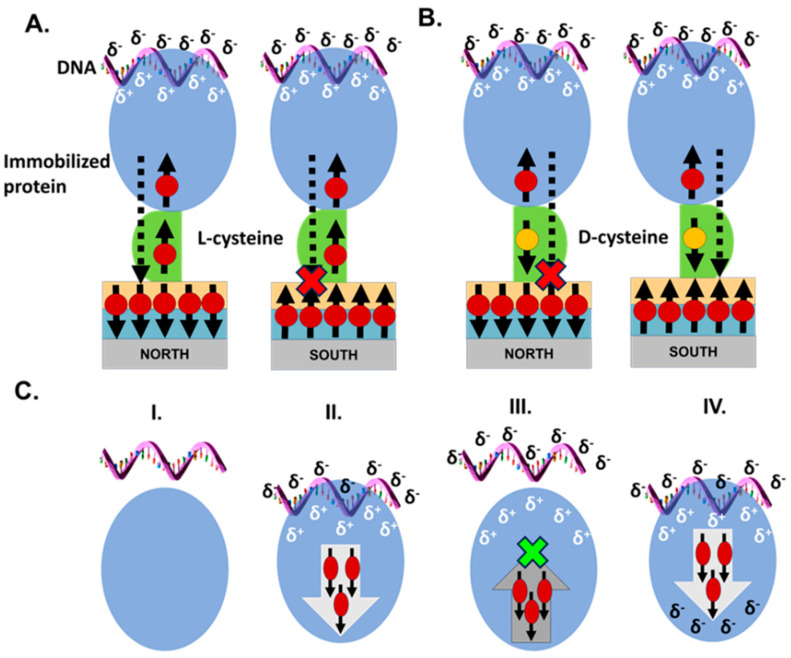
(**A**) Schematic representation of the mechanism of the effect of spin on protein–DNA interaction. When the spin on the ferromagnet is pointing opposite to the momentary spin of the charge at the interface of the protein, which is adsorbed on the surface through *L*-cysteine (representative spins and electrons migrating through the protein are shown as black arrows and red balls, respectively), charge flows more efficiently between the protein and the ferromagnetic substrate (shown as a dotted arrow penetrating the substrate). This scenario manifests when the North pole of the magnet is facing the ferromagnetic substrate. When the South pole of the magnet is facing the ferromagnetic substrate, the spins on the protein and the substrate point in the same direction, resulting in inefficient electron flow. (**B**) The effect of a *D*-cysteine linker on protein–DNA interaction. The terminal *D*-cysteine linker, shown in green, which has opposing chirality to the rest of the protein, flips the direction of spins transmitted through the protein (black arrow, yellow ball). Consequently, the opposite magnet polarity is required to achieve electron flow relative to the schematic in A. (**C**) Model describing the role of electron spin-related polarizability in the docking of a DNA molecule on a protein. The reaction scheme describes the dynamic interaction of a protein molecule with a DNA molecule to form a transient (protein–DNA) complex (charge-reorganization step) and the final energetically favored (protein–DNA) configuration (binding step). (**I**) The system before DNA–protein interaction. (**II**) The approaching DNA repels electrons with specific spins away from the binding zone. (**III**) Upon retraction of the DNA from the protein, the electrons that were withdrawn are not able to flow back because they have the “wrong” spin direction. (**IV**) The final docking that results in the binding of the DNA to the protein. The figure was copied with permission from reference [34].

An interesting role of electron spin is in controlling the relaxation of the induced dipole during an association reaction. In the process of protein–ligand docking, the ligand might be considered as approaching and retreating relative to the protein at a high frequency. This process may involve several stages, with a range of timescales. One may imagine that if the timescale of the process is very slow, the induced charge will be relaxed and will be rebuilt only when the ligand approaches the protein again. However, the situation can be very different for very rapid processes. Let us assume that a negatively charged DNA molecule is approaching a binding site on a protein and is inducing positive charge accumulation at this site. Electrons are then repelled from this site on the protein to other parts of it. Since the protein is chiral, the repelled charge is spin-selective. This charge cannot flow back to the binding site till the spins relax since flowing back requires flipping the spin direction (see Figure 3). Hence, the binding site remains positively charged even if the DNA molecule retreats during the docking process. This results in an increase in the rate of the docking process and finally in more efficient docking [34]. This hypothetical mechanism was modeled using relatively simple structures, but it should be probed in more detail experimentally and theoretically.

### 3.3. Modulation of Enzymatic Activity

It is well established that enzymatic activity can be modulated by light, typically due to the involvement of (potentially designed) conformational changes [35]. We recently found that polarizability effects can also be involved in mediating the effect of light on an enzyme. In our experiment, charge injection into a protein molecule was induced by a photosensitizer, which was attached to a predetermined position on protein molecules adsorbed on a surface, which did not have to be conductive or magnetic. The enzymatic activity of the protein was monitored as a function of the excitation of the dye by light, and to explore the spin dependence of the effect, circularly polarized light was used (Figure 1C).

**Figure 4:**
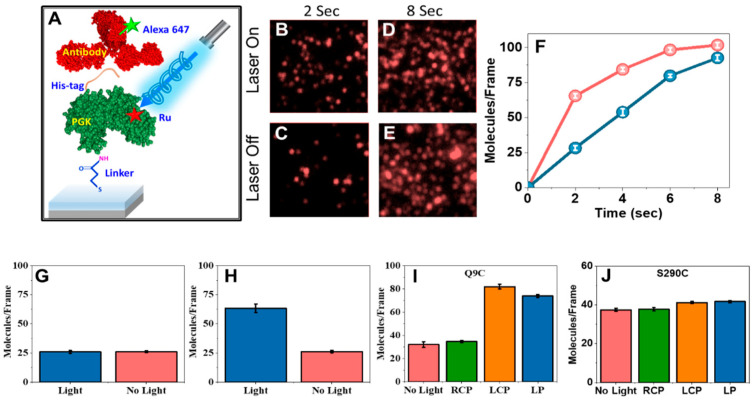
Modulating PGK–antibody interaction kinetics by photoexcitation. (**A**) Schematic of the experimental setup to study the effect of linearly and circularly polarized light on the interaction kinetics of a His-tagged ruthenium-photosensitizer modified PGK with an anti-His antibody. (**B**–**E**) Fluorescent images of individual complexes formed between His-tagged PGK molecules labeled with the photosensitizer at residue 9 and adsorbed on a gold surface and alexa-647-labeled anti-His antibodies in the presence and absence of illumination with linearly polarized light for 2 s and 8 s. (**F**) Kinetics of PGK–antibody interaction with (red) and without (blue) illumination, as measured by counting molecules in fluorescent images. (**G**) No effect of illumination on PGK–antibody interaction kinetics was observed in the absence of the photosensitizer. (**H**) The experiment shown in panels B-E was repeated with PGK adsorbed on glass, with similar results. (**I**) The effect of the polarization of the light on the photoinduced enhancement of PGK–antibody interaction kinetics. RCP—right circular polarization; LCP—left circular polarization; LP—linear polarization. (**J**) Only a minor illumination effect was observed when the photosensitizer was moved to residue 290. In (**G**–**J**), molecules were counted 2 s following the initiation of the reaction. At least nine regions were counted in each sample.

We used the enzyme phosphoglycerate kinase (PGK) here and attached a photosensitizer to two different sites on the protein. The excitation of the photosensitizer on the surface-immobilized molecules of the enzyme led to the injection of charges into it. We observed a surprising reduction in enzymatic activity by up to a factor of three, which depended on the polarization of the laser light used for excitation. A maximal reduction in enzymatic activity was found when the light was circularly polarized in one sense, while the opposite sense did not lead to a measured effect. These results are shown in Figure 4.

We conjectured that the suppression of enzymatic activity depends on the charge-injection position on the protein, as the transfer of charges through the protein matrix may depend on spin through the CISS effect. Indeed, moving the photosensitizer modulated the effect. However, a clear connection between the photosensitizer attachment site on the enzyme and the modulation of enzymatic activity has yet to be established. Clearly, exciting the photosensitizer changes the polarizability of the protein, which might affect the association of substrates with the enzyme’s active site. The ability to affect enzymatic activity by photoexcitation should allow us to map how information can be transferred between different sites on the protein, affected by its polarizability. Importantly, this effect can in principle be used to generate enzyme versions whose activity is controlled by photoexcitation.

## 4. Conclusions and Future Perspective

As shown above, the polarizability of proteins can serve as an allosteric signal, i.e., as a means of transferring information between different sites. Thus, changing either the structure or electronic state at one site may affect another site at a large distance. Changes in polarizability are communicated very fast within protein molecules, and if there is a significant electronic coupling between remote sites, polarizability is an efficient way to couple them. Because of their chirality, the polarizability of proteins is spin-dependent, with the spin acting as another degree of freedom for transferring information across them [21,25,26].

Understanding the effect of polarizability on the function of proteins requires mapping “communication paths” in proteins, namely, how an electrostatic modification at one site may propagate to another site. Currently, there are no theoretical or computational methods that can provide an answer to this question, such as those used previously to study electron transfer pathways [32]. Experimental data and their analysis should form a basis to start obtaining insights into the capability of different paths in a protein to transfer information using polarization effects and to decipher what makes a particular path efficient. This knowledge, together with advances in calculations, may enable us to use the electronic polarizability-related allosteric effect to design new types of enzyme-related devices.

An interesting concept that relates to the polarizability effect is the role of non-active “silent parts” in proteins. Many explanations have been introduced for the role of “inactive parts” of proteins. It was proposed that such parts affect the structural stability, the impact of mutations, and the kinetics of the interaction between two proteins, as well as between proteins and ligands [36,37,38]. An additional potential explanation for large protein sizes relates to the concept of charge-reorganization allostery. The size of a protein affects its polarizability and hence its ability to undergo strong electrostatic interactions. Since polarizability is a global property of proteins, affected by all their components, “silent parts” contribute to the polarizability and may affect reactivity in this manner. This conjecture remains to be further explored.

In summary, in this review, we addressed the possible importance of spin-dependent polarizability in the activity of proteins. This concept is new, and much more experimental and theoretical work must be performed before its role can be fully appreciated.
